# Successful bailout of complications during hot balloon ablation

**DOI:** 10.1016/j.hrcr.2022.08.004

**Published:** 2022-08-12

**Authors:** Masanaru Sawada, Koichi Nagashima, Yuji Wakamatsu, Naoto Otsuka, Satoshi Hayashida, Shu Hirata, Moyuru Hirata, Sayaka Kurokawa, Yasuo Okumura

**Affiliations:** Division of Cardiology, Department of Medicine, Nihon University School of Medicine, Tokyo, Japan

**Keywords:** Hot balloon, Atrial fibrillation, Complications, Pulmonary vein injury, Coronary air embolism

**Key Teaching Points**

• Complications during hot balloon ablation (HBA) such as pulmonary vein (PV) injury and air embolisms are rare but potentially fatal. Our cases highlighted the importance of an immediate diagnosis and bailout strategy.
• Once hemoptysis occurs during HBA, the electrophysiologists should be aware of any PV injury, and the immediate use of noninvasive positive pressure ventilation should be considered as a bailout strategy.
• When we encounter massive air on the fluoroscopic image and ST elevation during HBA, we should perform an emergent coronary angiography as soon as possible. A thrombus aspiration device may be an alternative option when mechanical aspiration via a coronary guiding catheter has failed.

## Introduction

The hot balloon (HB; Toray Industries, Inc, Tokyo, Japan) is one of the widely used balloon ablation technologies for the treatment of atrial fibrillation (AF) in Japan, and the balloon size can be adjusted to occlude the pulmonary vein (PV) ostia. With the increased use of this balloon ablation technology, complications have rarely occurred during HB ablation (HBA). We experienced 2 cases that had severe complications that were successfully resolved by a specific bailout strategy. This report aimed to share these experiences and bailout treatments.

## Case report

### Case 1: PV injury and hemorrhage

**Figure 1:**
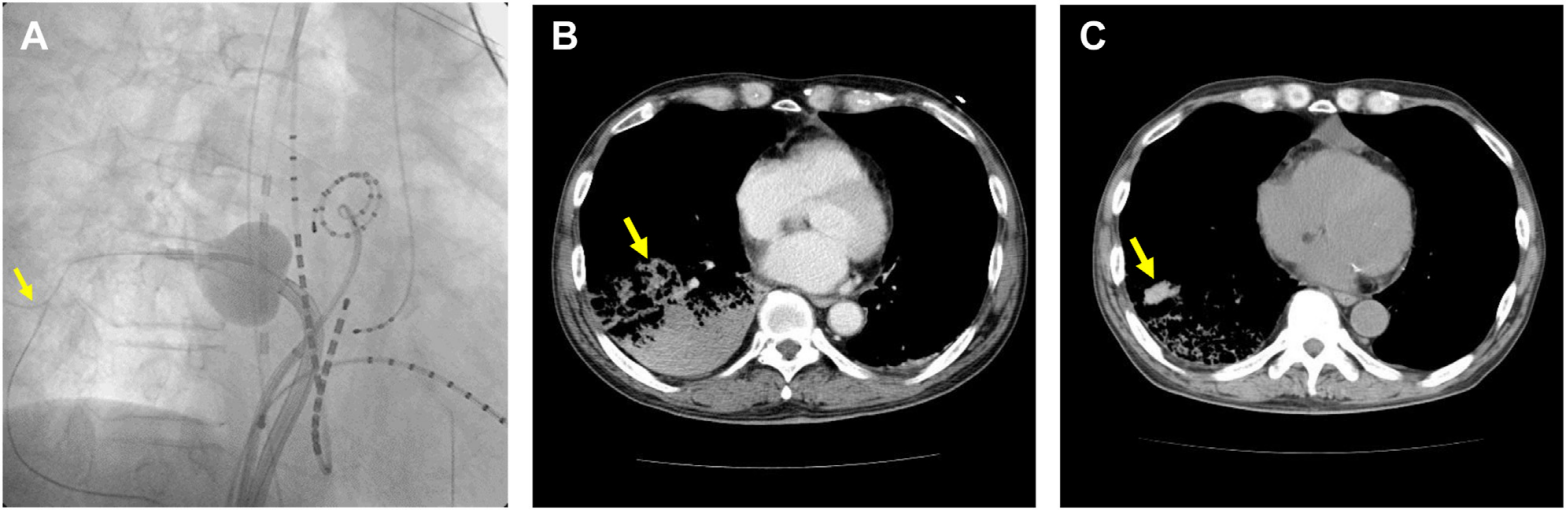
**A:** Fluoroscopic image during hot balloon ablation (HBA) of the right inferior pulmonary vein (RIPV). The yellow arrow indicates the guidewire advanced in the small branch of the RIPV in the fluoroscopic image. **B, C:** Computed tomography images immediately after (B) and 6 days after the HBA procedure (C). The yellow arrow in image B indicates the consolidation in the right lower lobe suggestive of a pulmonary hemorrhage. The yellow arrow in image C indicates that the pulmonary hemorrhage in the right lower lobe had improved.

A 75-year-old man with persistent AF received an HBA. The procedure was performed under conscious sedation with dexmedetomidine hydrochloride. After the right inferior PV isolation with the guidewire advanced deep in the PV, massive hemoptysis occurred, followed by a blood pressure reduction from 120 mm Hg to 90 mm Hg and hypoxia (arterial oxygen partial pressure of 80 mm Hg and delivered O_2_ 12 L/min via a reservoir face mask). Those vital signs did not recover despite a large-volume infusion and oxygen administration with a self-inflating bag. According to the fluoroscopic image with the guidewire peripherally positioned in the PV, we suspected the occurrence of PV injury (Figure 1A). Because his shock and hypoxia persisted after a considerable additional-volume infusion and a blood transfusion including 4 units of red blood cells and 2 units of frozen plasma, we decided to perform noninvasive positive pressure ventilation (NPPV) in a continuous positive airway pressure mode with a positive end-expiratory pressure of 4 cmH_2_O, which was titrated to 7 cmH_2_O. The hypoxia immediately recovered with the NPPV, and his blood pressure gradually increased up to 120 mm Hg. An emergent computed tomography scan after the procedure revealed extravasation of the right inferior PV branch and a massive infiltration suggestive of a hemothorax of the right lower lobe (Figure 1B). Two days later, the patient was weaned from the NPPV. His clinical course was favorable, without any episodes of hemoptysis after the direct oral anticoagulants were resumed. A computed tomography reevaluation on the sixth day after the procedure indicated a considerable improvement in the hemothorax (Figure 1C). He was discharged the next day.

### Case 2: Coronary air embolism

**Figure 2:**
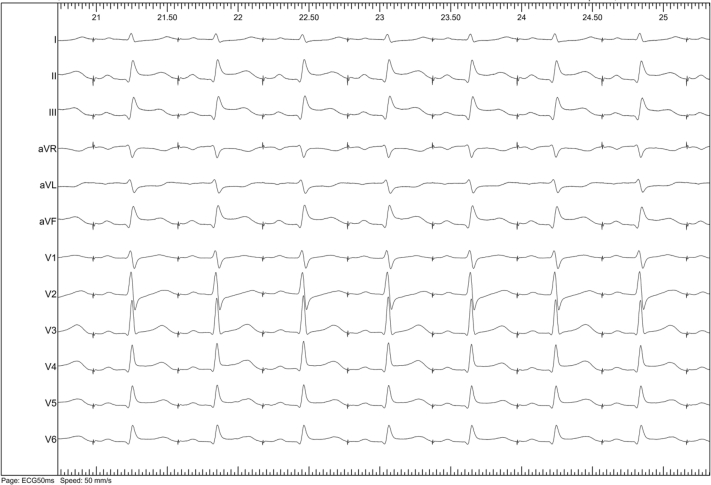
The 12-lead electrocardiograms during the catheterization revealed ST elevation in the inferior leads.

**Figure 3:**
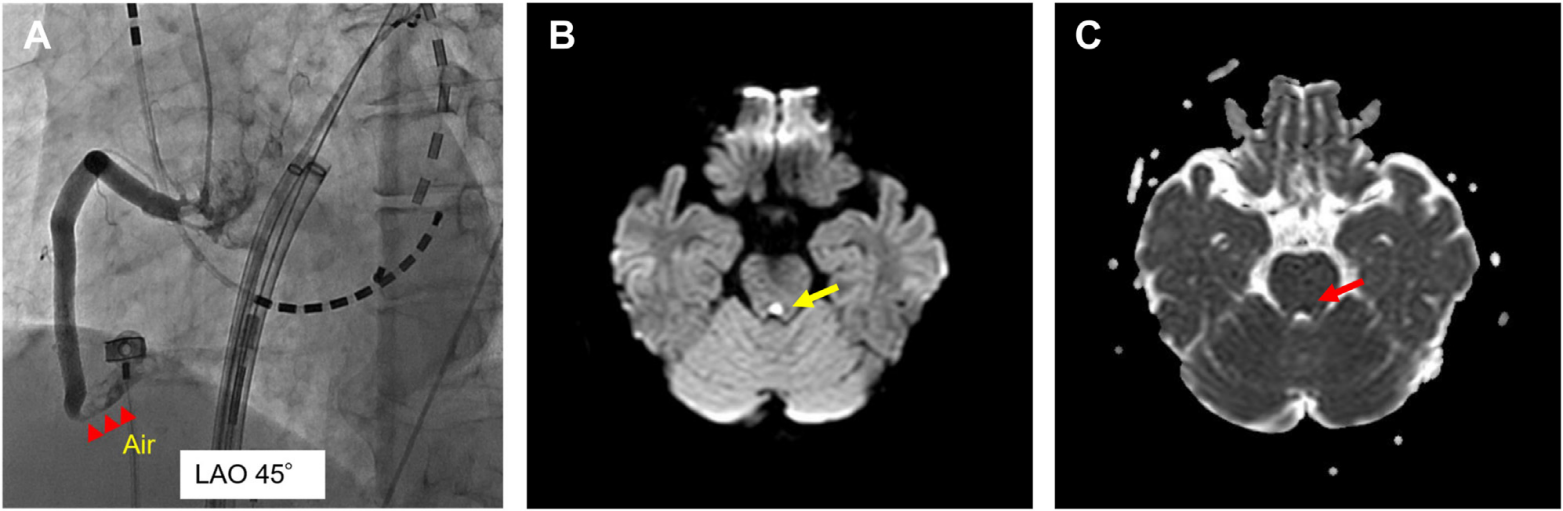
**A:** During the insertion of the balloon through the guiding sheath (Treswaltz; Toray Industries, Inc, Tokyo, Japan), angiography showed a right coronary artery occlusion due to massive air bubbles (*red arrowheads*). LAO = left anterior oblique. **B:** Diffusion-weighted magnetic resonance imaging of an acute stroke shows an infarct in the pontine tegmentum (*yellow arrow*). A low signal is seen on the apparent diffusion coefficient map (red arrow).

A 75-year-old woman with paroxysmal AF underwent an HBA. After the transseptal approach, the HB was inserted into the left atrium through a 13F deflectable guiding sheath (Treswaltz; Toray Industries). During the occlusion of the left superior PV, ST elevation was detected on the inferior leads (Figure 2) and she simultaneously experienced chest pain. A fluoroscopic image showed massive air in the right coronary artery (RCA) area; therefore, we performed an emergent coronary angiography within 5 minutes that revealed the air was trapped in RCA#3 (Figure 3A). We attempted to aspirate the air manually through a 5F Judkins R catheter but it failed. Therefore, a thrombus aspiration device (Thrombuster III SL; Kaneka Medix Corp, Osaka, Japan) was inserted over a guidewire to the site of the trapped air in the RCA. All visible air was successfully retrieved by the Thrombuster, and the ST elevation recovered without any Q waves. No ventricular arrhythmias associated with acute ischemia were observed. Given the complication, the PV isolation was performed with a standard radiofrequency energy catheter–based ablation. The next day, she experienced diplopia, nausea, and a bilateral ocular adduction disorder. Emergent magnetic resonance imaging revealed a small acute cerebral infarction in the pontine tegmentum (Figure 3B). Taking into account her clinical symptoms, she was diagnosed with left medial longitudinal fasciculus syndrome. Because the infarction lesion was relatively small, hyperbaric oxygen therapy was not attempted. A direct oral anticoagulant was continued during the hospitalization. The bilateral ocular adduction disorder partially recovered with eye movement rehabilitation, and she was discharged on the 22nd day and had completely recovered by 3 months later.

## Discussion

### Case 1: PV injury and hemorrhage

During the balloon-based ablation, it has been reported that guidewire-related complications are rarely caused by a guidewire being positioning deep in the distal PV or left atrial appendage, as manifested by a PV hemorrhage or LA perforation.^1^^,^^2^ The incidence is reported to be 0.35% during cryoballoon ablation, but it is unknown during HBA.^3^ Electrophysiologists should always be aware of the position of the guidewire tip while advancing the balloon to prevent any PV injury or left atrial appendage perforation. Because a relatively stiff-tip 0.032-inch-diameter guidewire is generally used for HBA to establish a good balloon-to-tissue contact, the wire may have contributed to the PV injury. The incremental increase in the intrathoracic and alveolar pressure and decrease in the venous return owing to the NPPV might have decreased the bleeding that was contributed to by the pressure gradient between the PV and alveoli. In addition, the NPPV might have improved the oxygenation owing to the inflation of the atelectasis by the hemothorax. Because the NPPV has the risk of further reducing the blood pressure owing to the decrease in the venous return, hemostasis would be the priority treatment. Once hemoptysis occurs during HBA, the electrophysiologists should be aware of PV injury, and the immediate use of NPPV should be considered as a bailout strategy. If this occurs under general anesthesia, a higher positive end-expiratory pressure may be an option.

### Case 2: Coronary air embolism

The HRS/EHRA/ECAS/APHRS/SOLAECE expert consensus statement reported the incidence of air embolisms is less than 1% and cerebral infarctions/transient ischemic attacks 0–2%.^4^ Air embolisms are generally known to occur during the insertion and removal of catheters from the sheath. In particular, air embolisms are rare but are slightly more prevalent during balloon-based ablation than standard catheter-based ablation because of the use of large sheaths. Miyazaki and colleagues^5^ reported that the sole clinically manifesting air embolisms were coronary air embolisms, and they occurred in 2.6% of patients who underwent cryoballoon ablation. All were related to the transseptal sheaths during the early phase of the procedure, but all patients recovered without any sequelae under medical management. They proposed that the insertion of the CB into the sheath in a water bucket, followed by air aspiration while in the sheath with a syringe, tended to reduce the incidence; however, air embolisms can still occur.^5^ A common presentation of an air embolism is ST-segment elevation in the inferior leads because the RCA is the most superior coronary branch when the patient is in the supine position. This embolism leads to an atrioventricular block and cardiogenic shock. In our case, the air trapped in the RCA (#3) could be removed only by a thrombus aspiration device (Thrombuster III SL; Kaneka) inserted over a guidewire to the site of the trapped air in the RCA. Therefore, aspiration at the local site in the coronary artery with the thrombus aspiration device may be an alternative option when mechanical aspiration via a coronary guiding catheter has failed. To prevent air embolisms, persistent efforts to maintain an airtight sheath condition are also important.

## Conclusion

Both PV injury and air embolisms as complications during HBA are rare but potentially fatal. Our cases highlighted the importance of an immediate diagnosis and a bailout strategy that can potentially resolve those complications.
